# Encapsulation of Anti-Tuberculosis Drugs within Mesoporous Silica and Intracellular Antibacterial Activities

**DOI:** 10.3390/nano4030813

**Published:** 2014-09-11

**Authors:** Xin Xia, Kevin Pethe, Ryangyeo Kim, Lluis Ballell, David Barros, Jonathan Cechetto, HeeKyoung Jeon, Kideok Kim, Alfonso E. Garcia-Bennett

**Affiliations:** 1 Department of Materials and Environmental Chemistry, Arrhenius Laboratory, Stockholm University, Stockholm SE-106 91, Sweden; E-Mail: xin.xia@mmk.su.se; 2 Nanologica AB, Drottning Kristinas Väg 61, Stockholm SE-114 28, Sweden; E-Mail: xin@nanologica.com; 3 Screening Technologies and Pharmacology Group, Institute Pasteur Korea, Bundang gu, Seongnam-si, Gyeonggi-do 463-742, Korea; E-Mails: kevin.pethe@ip-korea.org (K.P.); rykim@ip-korea.org (R.K.); jonathancechetto@gmail.com (J.C.); hkhk79@hotmail.com (H.J.); kideok.kim@ip-korea.org (K.K.); 4 The Diseases of the Developing World Centre, GlaxoSmithKline, Severo Ochoa 2, Tres Cantos, Madrid 28760, Spain; E-Mails: lluis.p.ballell@gsk.com (L.B.); david.a.barros@gsk.com (D.B.)

**Keywords:** tuberculosis, mesoporous materials, solubility, intracellular, formulation, nanomedicine

## Abstract

Tuberculosis is a major problem in public health. While new effective treatments to combat the disease are currently under development, they tend suffer from poor solubility often resulting in low and/or inconsistent oral bioavailability. Mesoporous materials are here investigated in an *in vitro* intracellular assay, for the effective delivery of compound PA-824; a poorly soluble bactericidal agent being developed against Tuberculosis (TB). Mesoporous materials enhance the solubility of PA-824; however, this is not translated into a higher antibacterial activity in TB-infected macrophages after 5 days of incubation, where similar values are obtained. The lack of improved activity may be due to insufficient release of the drug from the mesopores in the context of the cellular environment. However, these results show promising data for the use of mesoporous particles in the context of oral delivery with expected improvements in bioavailability.

## 1. Introduction

Tuberculosis (TB) is a lethal infectious disease that remains a major problem in public health. According to the World Health Organization (WHO) more than 2 billion people are infected by Mycobacterium tuberculosis (MTB), and 1.3 million people died from TB in 2012 [1]. Treatments of tuberculosis have been established since the 1940s. However, current therapies have limited efficacy against drug-resistant tuberculosis. MTB is highly susceptible to mutations and easily develops drug resistance [2]. New treatments with shorter regimes, which are effective against drug resistant strains, are the current focus of anti-tuberculosis research. For instance, the nitroimidazole PA-824 was reported to act against drug-resistant and drug-susceptible TB [3]. According to a recent *in vivo* study using a TB mice model, PA-824 has a bactericidal activity slightly greater than the standard first line drug rifampicin (20 mg/kg), and comparable to moxifloxacin (100 mg/kg) and isoniazid (25 mg/kg) [4]. Nuermberger *et al.* [5] reported that a new regime containing PA-824, moxifloxacin and pyrazinamide shortened the treatment in mice compared to the combination of rifampin, isoniazid and pyrazinamide. In a 14-day study conducted by Diacon *et al.* [6], it was found that the combination of PA-824, moxifloxacin, and pyrazinamide has higher efficacy than that of bedaquiline, bedaquiline-pyrazinamide, bedaquiline-PA-824; and similar to the current standard regimes for drug-susceptible tuberculosis on TB patients. This is important for the treatment of patients that are less sensitive to standard regimes.

Although PA-824 has a high anti-tubercular activity, it suffers from poor water solubility that limits the bioavailability and limits the formulation to non-oral administrations. A traditional solution for poor solubility issues is to increase the surface area of the drug crystals by reducing their size through for instance micronization, or using solubilizers such as surfactants. However, these methods are typically not economically viable or not efficient enough in enhancing the solubility of the drug via a supersaturated state, and hence its bioavailability [7]. Li *et al.* [8] reported recently that changing the inherent lattice stability by altering different chemical groups in the PA-824 molecule was not able to increase its aqueous solubility. Hence, the formulation of PA-824 remains an important unresolved problem. Poor bioavailablity may contribute to patient non-compliance issues and the risk of overdosing in oral therapies [9,10]. The hepatotoxicity associated with more potent anti-tuberculosis drugs due to their poor solubility and high required dosage, can sometimes limit their further development [11].

Mesoporous silica have been reported to enhance the solubility of several poorly water-soluble drugs with a variety of physico-chemical profiles [12,13,14], making them promising formulating agents for novel antituberculars. Clemens *et al.* [15], utilized mesoporous silica nanoparticles with a polyethyleneimine coating to release rifampin and with cyclodextrin-based pH-operated valves that open only at acidic pH to release isoniazid into acidified *M. tuberculosis*-infected macrophages. Both material designs were significantly more effective than an equivalent amount of free drug in their assay. Mesoporous nanpparticles capped by ε-poly-l-lysine cationic polymer have been reported to enhance the efficacy of antimicrobial drugs [16]. Mesoporous materials are a family of nanostructured particles characterized by ordered pores or cages with diameters in the range 2–50 nm. Since 2001, they have been studied as candidates for a variety of pharmaceutical applications [17]. Ordered mesoporous silica particles have been additionally shown *in vitro* and *in vivo* to: load large pay loads of single or multiple active molecules [12]; tailor the pharmacokinetic release profiles through diffusion or other mechanisms [18]; target the release of pharmaceutical products to specific tissues [19]; increase the bioavailability of pH sensitive drug candidates [13,20]; act as adjuvants in immunotherapies [21]; act as a diagnostic and theranostic particles [22]; and enhance the growth of apatite layers in tissue generation and bone implants [23].

There are a number of inherent physical properties of mesoporous materials which make them attractive for drug delivery applications: their high surface area (above 1000 m^2^/g), large pore volumes (~1 cm^3^/g), sharp and controllable pore size distributions, their chemically and thermally stable compositions and the low associated toxicity [24]. The combination of these properties results in a very versatile excipient for drug development. Witasp *et al.* [25] have reported that mesoporous silica particles of average size of 300 nm show an efficient internalization by primary human macrophages without impairment of the cell viability or macrophage function. In their intracellular study, calcined mesoporous particles with cubic 3D-cylindrical pores of 4.0 nm in average size, at 100 µg/mL concentration, were presented to macrophages for 1 and 6 h. Both time-points showed massive internalization of mesoporous particles. Since macrophages are the main host cell of MTB, where the mycobacteria invade and replicate [26], a delivery system that could reach the macrophage and release the anti-tuberculosis drugs locally would increase the efficacy of the drug by achieving higher concentration at the infected area.

**Figure 1:**
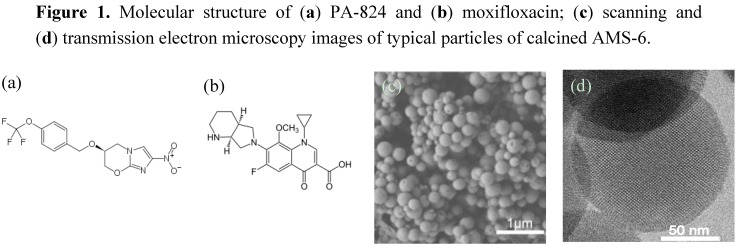
Molecular structure of (**a**) PA-824 and (**b**) moxifloxacin; (**c**) scanning and (**d**) transmission electron microscopy images of typical particles of calcined AMS-6.

Herein, we utilize mesoporous silica (Figure 1, with the same physical properties as particles used by Witasp *et al.* [25]) as a drug delivery vehicle for the solubility enhancement of PA-824. To better understand the mode of action of mesoporous particles, and for comparison, we also encapsulated moxifloxacin into the same mesoporous silica particles. Moxifloxacin is a commercially used water-soluble anti-tuberculosis drug under brand names Avelox, Avalox and Avelon for oral TB treatment. We show *in vitro* release profiles of both drugs as well as performing intracellular assays (Scheme I) on MTB-infected macrophages, with the aim of evaluating the antibacterial activity of the free and encapsulated drugs.

**Scheme I:**
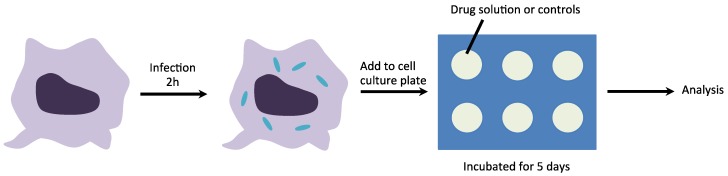
Scheme of the experimental design, silica particles loaded with PA-824 or moxifloxacin are presented to infected macrophages for the evaluation of the antibacterial activity of the free drug and encapsulated drug.

## 2. Results and Discussion

### 2.1. Loading and Dissolution Studies

AMS-6 mesoporous silica particles are spherical in shape with an average size of 300 nm in diameter as measured from SEM. Dynamic light scattering conducted in PBS shows peak maxima around 400 nm (see Figure S1). The pore size is 54.9 Å in a 3D-connectivity that gives a surface area 894 m^2^/g (Table 1). PA-824 and moxifloxacin are encapsulated into AMS-6 particles by solvent evaporation using ethanol as solvent (see experimental section for more details).

**Figure 2:**
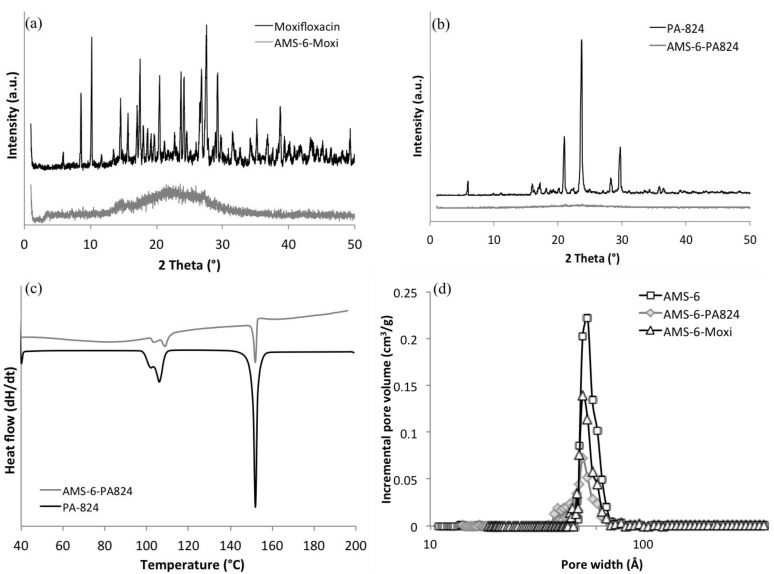
X-ray diffraction patterns of (**a**) free moxifloxacin, and AMS-6-Moxi; (**b**) free PA-824, and AMS-6-PA824. Both show that the free drug in its crystalline state and the encapsulated material contains drug in the amorphous state; (**c**) differential scanning calorimetry (DSC) curves of free and loaded PA-824; (**d**) pore size distribution of calcined AMS-6, AMS-6-PA824, and AMS-6-Moxi obtained from density functional theory (DFT).

The loading amount is 28.0 wt% and 40.4 wt%, respectively, determined by thermogravimetric analysis (TGA) and calculated from the decomposition temperature between 200 and 900 °C (see Figure S2). The measured decomposition temperature of free PA-824 was 307 and 287 °C for the encapsulated PA-824. A lower decomposition temperature for the encapsulated compound may be indicative of a transformation of the compound from crystalline form (free form) to the amorphous form upon loading into the pores. X-ray diffraction curves (Figure 2) show that no recrystallization occurs in the encapsulated PA-824 and moxifloxacin, whilst the free drug is highly crystalline. However, small peaks are observed in the differential scanning calorimetry (DSC) curve of encapsulated PA-824, indicating that a small proportion of crystallization does occur during the loading procedure. No peaks at melting temperature were observed in DSC curve of moxifloxacin therefore it is loaded in an amorphous state (see Figure S3). A comparison of textural properties from nitrogen adsorption isotherms of encapsulated and calcined materials allows us to calculate the extent of pore filling through a decrease in pore volume.

**Table 1:** Textural properties of calcined and encapsulated AMS-6 particles.

Samples	Surface area (m^2^/g)	Pore width (Å)	Total pore volume (cm^3^/g)	Loading amount (wt%)
AMS-6	894	54.9	1.07	-
AMS-6-PA824	513	52.0	0.55	28.0
AMS-6-Moxi	534	52.0	0.65	40.4

The BET surface area of mesoporous silica particles decreases from 894 to 513 m^2^/g after loading. The pore volume decreases from 1.07 to 0.55 cm^3^/g, which indicates that more than half of the pore space is occupied by PA-824. The BET surface area of encapsulated moxifloxacin decrease from 894 to 534 m^2^/g, and the total pore volume change to 0.648 cm^3^/g from 1.07 cm^3^/g. A comparison of the physical adsorption properties of encapsulated and calcined materials is shown in Table 1.

**Figure 3:**
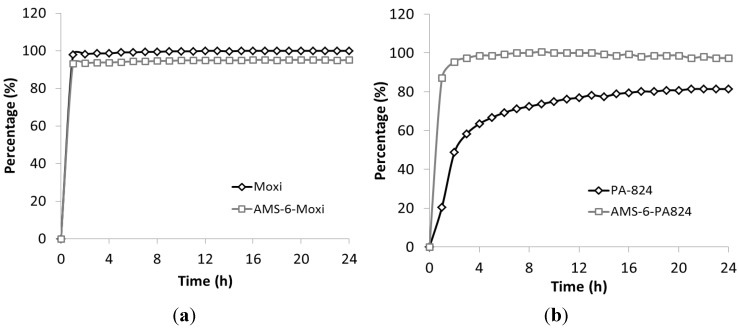
Kinetic release curves of (**a**) free and encapsulated moxifloxacin and (**b**) PA-824; in phosphate buffered saline buffer (PBS buffer, 10 mg/L).

*In vitro* dissolution tests were performed in phosphate buffered saline buffer (PBS) at pH 7.4 (Figure 3). Due to its significant solubility in water (168 mg/L), moxifloxacin shows a rapid dissolution rate for both encapsulated and free drug. The PA-824 free drug shows a slow release rate and poor solubility in PBS buffer due to its poor aqueous solubility. Encapsulated PA-824 shows a considerably faster dissolution rate than free drug, and it released 100% of the amount loaded within 4 h, whilst the free drug only release 63% after 4 h and 80% in 24 h. The solubility of PA-824 reaches 10 mg/L by encapsulation within AMS-6 mesoporous silica, which is 20% increase in comparison to the solubility of the free (crystalline) PA-824 as measured in this study (*ca.* 8 mg/L). No recrystallization occurs during the dissolution experiments.

The curves of free PA-824 can be fitted for both power law and the Higuchi models (Table 2) [27,28,29]. However, the encapsulated PA-824 showed a much faster initial release which is not consistent with a Higuchi diffusion-based mechanism of release. The enhancement in apparent solubility is related to the amorphous state of encapsulated PA-824 within the mesopores, in accordance to previous observed results [13]. Two kinds of interactions must be broken to dissolve a solute molecule: (i) the interactions among solute molecules in the crystal (lattice energy); and (ii) the interaction among the solvent molecules in the space require the accommodation of the solute molecule (cavitation energy) [30]. Therefore, most poorly water-soluble drugs show higher apparent solubility in an amorphous state since they require a lower energy barrier to dissolve compared to their crystalline counterparts.

**Table 2:** Parameters of power law and Higuchi equations for the release curves of free and encapsulated PA-824 (*k*: Kinetic constant; *n*: release exponent; *R*: coefficient of correlation, *t*_50%_: time taken to release 50% of PA-824).

Sample	Power law: ln*F* = ln*k*_p_ + *n*ln*t*			Higuchi: *F* = *k*_H_*t*_50%_^1/2^		
	*n*	*k*_p_	*R*^2^	*k*_H_	*t*_50%_ (h)	*R*^2^
PA-824	0.83	0.22	0.92	0.31	2.45	0.86
AMS-6-PA824	0.09	0.87	0.94	–	–	<0

### 2.2. Intracellular Assay

PA-824 against both multidrug-resistant and non-replicating MTB suggest that it may act by the inhibition of protein and lipid synthesis. The inhibition of an enzyme or deplete a cofactor that is responsible for the oxidation of hydroxymycolate to ketomycolate (cell wall) may be the mechanism of PA-824 [3]. Another study suggests that PA-824 may also act as intracellular nitric oxide donors which kills nonreplicating MTB [31].

To evaluate the intracellular efficacy of free and encapsulated drug, an assay was performed on Raw 264.7 macrophages expressing green fluorescent protein (GFP) and infected with MTB. In a previous study by Witasp *et al.* [21], AMS-6 particles were present to macrophages at 10 or 100 µg/mL, which is in the same range of our experiments. Their results suggest that macrophages could take up massive amounts of AMS-6 particles within one hour. The intracellular results present in this paper are based on the screening of macrophage growth and the inhibition of MTB.

**Figure 4:**
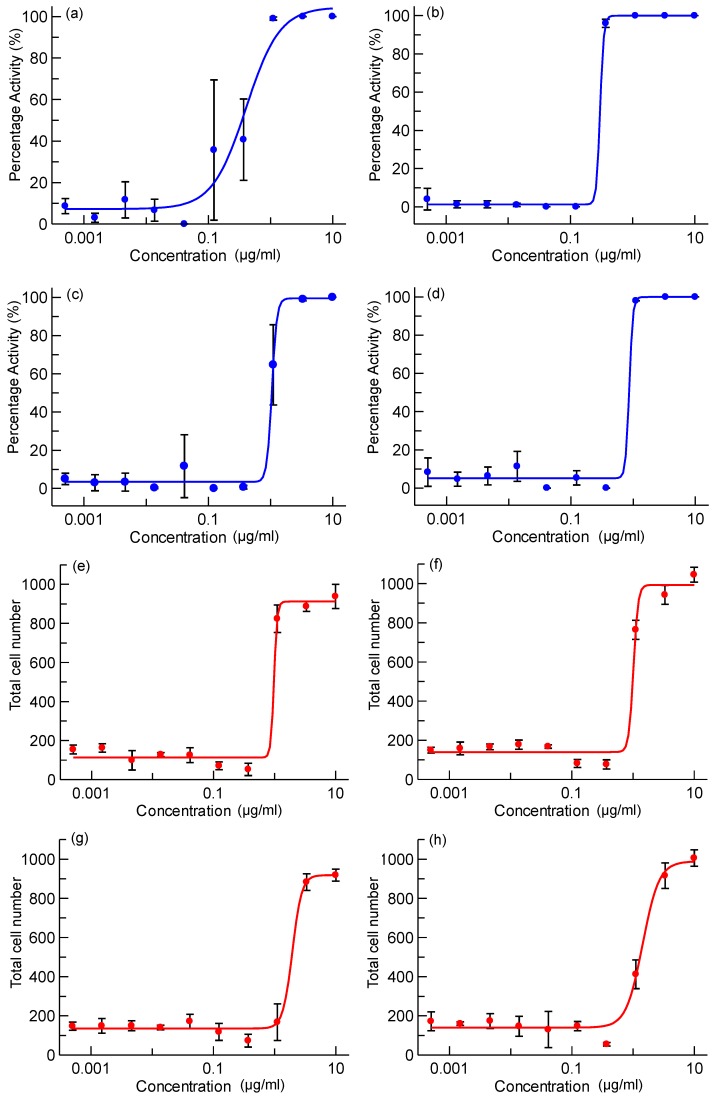
Antibacterial activity percentage of (**a**) PA-824; (**b**) moxifloxacin; (**c**) AMS-6-PA824; and **(d)** AMS-6-Moxi. Total macrophages number after present them to drugs in different concentrations: (**e**) PA-824; (**f**) moxifloxacin; (**g**) AMS-6-PA824; and (**h**) AMS-6-Moxi (Concentration in logarithm scale).

**Figure 5:**
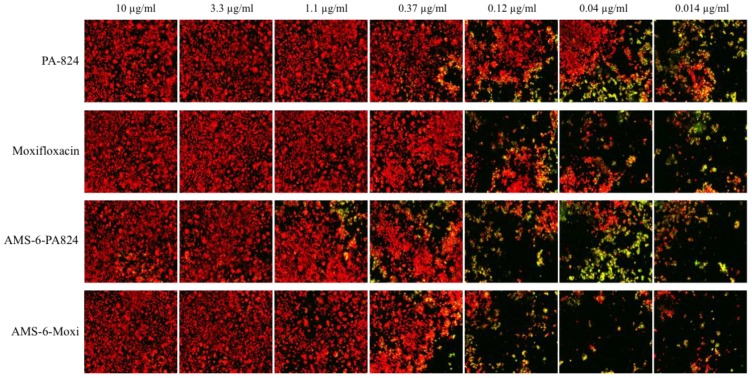
Confocal microscopy images of intracellular growth of tubercle bacilli inside macrophages under the treatment of PA-824, moxifloxacin, encapsulated PA-824, and encapsulate moxifloxacin at different drug concentration. Green-bacteria with green fluorescence; Red-alive macrophages.

Wells pre-plated with 0.5 mL of drug compounds, drug-loaded mesoporous silica particles or controls were dispensed with 10% heat-inactivated fetal calf serum supplement onto Raw 264.7 cells infected with M. tuberculosis (H37Rv-GFP) at a multiplicity of infection of 2:1 and dispensed into 384-well plates. After 5 days of incubation, macrophages were stained and measured for bacterial load and macrophage number (see experimental details). Figure 4 shows the percentage activity and total cell number of PA-824 and moxifloxacin (for both encapsulated and free compound). The antibacterial activity of free and encapsulated PA-824 shows a dose-dependent increase. Both free and encapsulated PA-824 reached the highest antibacterial activity at a concentration of 3.33 µg/mL. Free moxifloxacin reach the maximum activity at 0.37 µg/mL, whilst the encapsulated moxifloxacin reached its maximum activity at 1.11 µg/mL. Pure mesoporous silica particles did not show any anti-bacterial effect (see Figure S4). The total cell number counts provide additional information about the antibacterial activity and these are consistent in showing that both free and encapsulated PA-824 are active to MTB inside macrophages (Figure 5). The EC_50_ (half maximal effective concentration) values for compounds studied are 0.40, 1.03, 0.31 and 0.88 µg/mL for PA-824, AMS-6-PA824, moxifloxacin and AMS-6-Moxi, respectively.

Despite a significant enhancement in the solubility of PA-824 under kinetic release studies, overall evidence of an enhanced intracellular activity is not observed for the encapsulated drugs. Several reasons could be responsible for this lack of *translational effect*. Firstly, in the conditions of the cellular assay, the formation of protein coronas around the particles are likely to form, as has been found in a number of studies [25,32], which effects the release kinetics of the encapsulated PA-824 from within the pores, delaying the intracellular transport of drug molecules within the macrophages. Secondly, in the intracellular assay, macrophages and tested compounds were incubated for five days at 37 °C. The release of PA-824 in our study is in the same time range as the uptake of AMS-6 particles by macrophage. It is reasonable to assume that the majority of drug released intracellularly [31]. PA-824 also exhibits a time-dependent activity in a murine model [33]. In a pharamacokinetic study in human, the maximal plasma levels reaches in 4 to 5 h after oral administration, independent of dose [34]. Therefore, collection of kinetic information of antibacterial activity at intervals during these five days would fully determine if encapsulation has a faster antibacterial effect. Finally, measurements of the drug concentration remaining within the mesoporous particle after the intracellular incubation period would help understand the lack of improvement within our assay. However, it is important not to neglect the fact that the advantages of PA-824 release from mesoporous materials may be better observed in the context of an oral administration and *in vivo* study, where drug solubility may play a larger role [13]. As expected, free and encapsulated moxifloxacin did not show any difference in antibacterial activity as it is highly water-soluble (releasing straight away in the cell culture medium). Confocal microscopy images (Figure 5) show a trend of increase of macrophages (red) and decrease of MTB (green) with drug concentrations for all drugs in this work.

## 3. Experimental Section

### 3.1. Synthesis of AMS-6

The method used for AMS-6 synthesis has been described previously [35]. The surfactant, *N*-Lauroyl-*L*-Alanine (C_12_Ala, Nanologica AB, Stockholm, Sweden), was first dissolved in water, followed by addition of 3-aminopropyl triethoxysilane (APES, Sigma-Aldrich, St. Louis, MO, USA) which was used as a co-structure directing agent and tetraethyl orthosilicate (TEOS, Sigma-Aldrich) as silica source. All chemicals were used as received. In a typical synthesis, a homogenous solution of C_12_Ala in distilled water is kept at 80 °C for 24 h under static conditions. The surfactant solution was stirred for 10 min before addition of APES; TEOS was added 3 min after APES. The solution was stirred for another 15 min at 80 °C in a closed bottle. The synthesis gel was subsequently stored at room temperature (RT) under stirring for 24 h. The final synthesis mixture was kept sealed at 100 °C under static conditions for 3 days. The solid product was filtered and dried at RT and under atmospheric pressure conditions. The molar composition of the reaction mixtures was C_12_Ala:APES:TEOS:H_2_O = 1:1.25:6.7:309.1. Materials were calcined at 550 °C for 6 h. The materials were stored in airtight containers after calcination.

### 3.2. Thermogravimetric Analysis

Thermogravimetric analysis (PerkinElmer, Foster City, MA, USA) has been using to determine the loading amount of the encapsulated samples. The scanning was performed from 20 to 900 °C at a heating rate of 20 °C/min. The plug in gas atmosphere is dry air (flow rate is 20 mL/min). The weight of the samples is varied from 5 to 10 mg. The derivative weight loss was calculated by Pyris-instrument managing software.

### 3.3. Nitrogen Sorption Isotherm

Nitrogen adsorption-desorption isotherm (Micromeritics Tristar II 3020-Apparatus, Norcross, GA, USA) was used to investigate the porosity of calcined AMS-6 particles and the encapsulated particles. Calcined AMS-6 particles were degased at 300 °C for 6 h under nitrogen gas flow, and 8 h at 30 °C for loaded AMS-6. The surface area is calculated by the Brunauer-Emmett-Teller equation in the relative pressure range between 0.05 and 0.2 [36].

### 3.4. Media for Dissolution Experiments

Phosphate buffered saline (Sigma-Aldrich) used as a buffer was prepared by dissolving 1 PBS tablet in 200 mL water to give a pH of 7.4.

### 3.5. Loading of Pharmaceutical Active Ingredients to Mesoporous Silica

PA-824 is a free sample from TB Alliance (New York, NY, USA), and moxifloxacin is a free sample from GlaxoSmithKline (Tres Cantos, Spain). Drug compounds were loaded within the mesoporous silica particles via an evaporation technique followed by removal of the solvent using rotary evaporation. Typically, a concentrated solution of the drug was obtained in ethanol followed by direct addition of the calcined and dried mesoporous silica (200 mg) AMS-6. The slurry was stirred for 2 h, followed by solvent evaporation. Removal of the solvent was conducted at 45 °C under vacuum, before the recovered powder was dried overnight in air.

### 3.6. The Release Performance of Drugs in PBS Buffer

The *in vitro* release performance of drug loaded in ordered mesoporous silica was assessed in PBS buffer at pH 7.4. Five milligrams or equivalence of drug are added into 500 mL PBS buffer under a stirring rate of 100 rpm. All the release experiments showed good reproducibility. The release profile is obtained by means of UV-Vis absorbance scan (CE-3021, Cecil, Cambridge, UK).

### 3.7. M. tuberculosis Assay

The assay was performed as previously described [28]. Briefly, 384-well Evotec plates (#781058) were first pre-plated with 0.5 mL of drug compounds or drug-loaded mesoporous silica particles or controls dispensed by EVOBird (Evotec, Hamburg, Germany) in 10 µL RPMI 1640 (Gibco, Carlsbad, CA, USA) supplemented with 10% heat-inactivated fetal calf serum (FCS, Gibco). Raw 264.7 cells (American Type Culture Collection TIB-71) were infected with M. tuberculosis H37Rv-GFP at a multiplicity of infection of 2:1 and dispensed into 384-well plates. After 5 days of incubation, macrophages were stained with Syto 60, 5 µm (Invitrogen, S11342, Carlsbad, CA, USA) for 1 h at 37 °C. Image acquisition was performed on an EVOscreen Mark III platform integrated with Opera (Evotec, Hamburg, Germany). Bacterial load and macrophage number were quantified using proprietary image analysis software. Isoniazid and DMSO has been used as positive and negative controls, respectively.

## 4. Conclusions

Encapsulation of PA-824 into mesoporous particles AMS-6 results in an enhancement in the solubility and rate of PA-824 *in vitro* kinetic release studies. In the present intracellular assay, mesoporous silica showed similar antibacterial activity as the free drug after 5 days of incubation in macrophage TB-infected cells. Mesoporous silica on its own did not show any antibacterial properties. The lack of improvement in antibacterial activity is likely due to insufficient release of the drug from within the pores and the lack of kinetic release results. The intracellular kinetic release of PA-824 after the particles have been engulfed by macrophages is of great interest and worth to investigate, which is also relevant for other diseases involving intracellular delivery. Further work will focus on understanding in more depth the kinetic effects associated with macrophage uptake and release through the use of fluorescent probes, as well as in the *in vivo* confirmation of antibacterial activity in suitable animal model and through oral administration, where mesoporous AMS-6 particles are likely to show direct benefit due to improvements in the bioavailability in comparison to the free PA-824 drug.

## Supplementary Materials

Supplementary File 1

## Supplementary Materials

Supplementary materials can be accessed at: http://www.mdpi.com/2079-4991/4/3/813/s1
