# Supplementary material for: Encapsulation of Anti-Tuberculosis Drugs within Mesoporous Silica and Intracellular Antibacterial Activities
Source: Nanomaterials (Basel). 2014 Sep 11;4(3):813–26. doi: 10.3390/nano4030813 (PMC5304699; doi:10.3390/nano4030813)
Supplement: Supplementary File 1 [file nanomaterials-04-00813-s001.pdf]

Supporting Information

Figure S1. Particle size distribution of calcined AMS-6 particles in PBS (Obtained from dynamic light scattering).

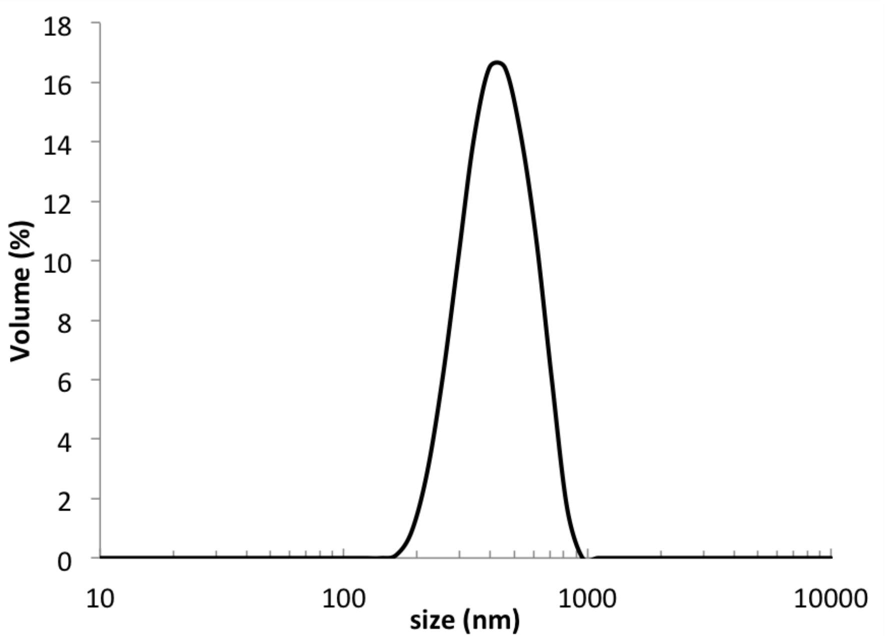

Figure S2. TGA curves of (**a**) PA-824; (**b**) AMS-6-PA824; (**c**) moxifloxacin; and (**d**) AMS-6-Moxi.

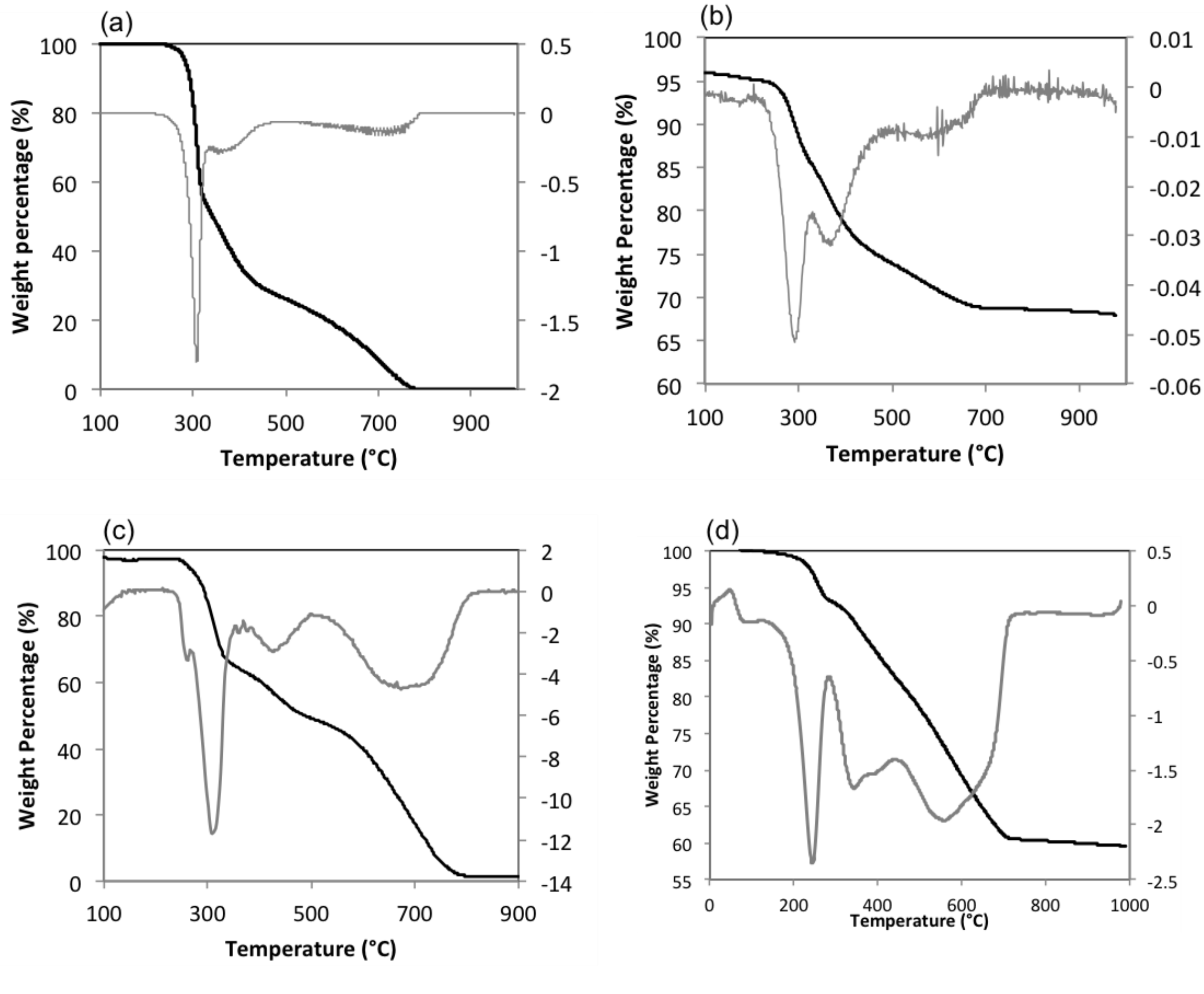

Figure S3. DSC curves of free and loaded moxifloxacin.

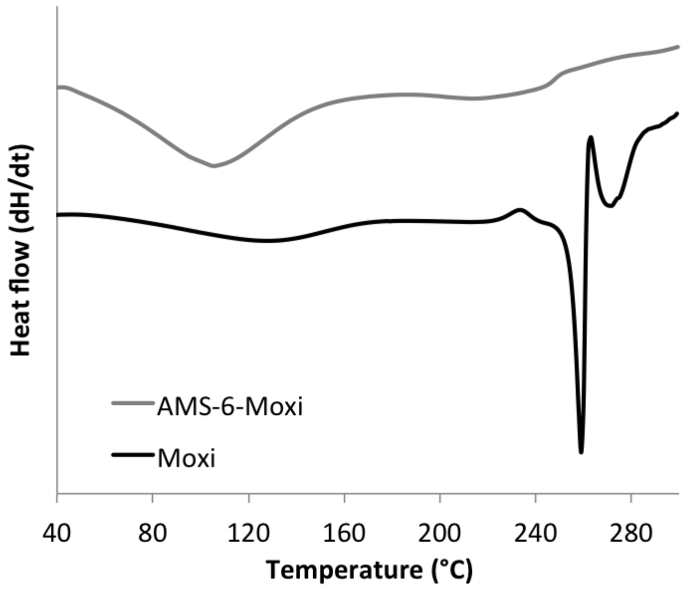

Figure S4. (**a**) Antibacterial activity percentage of calcined AMS-6 particles; (**b**) total macrophage number after present to calcined AMS-6 particles. (Concentrations are in logarithm scale).

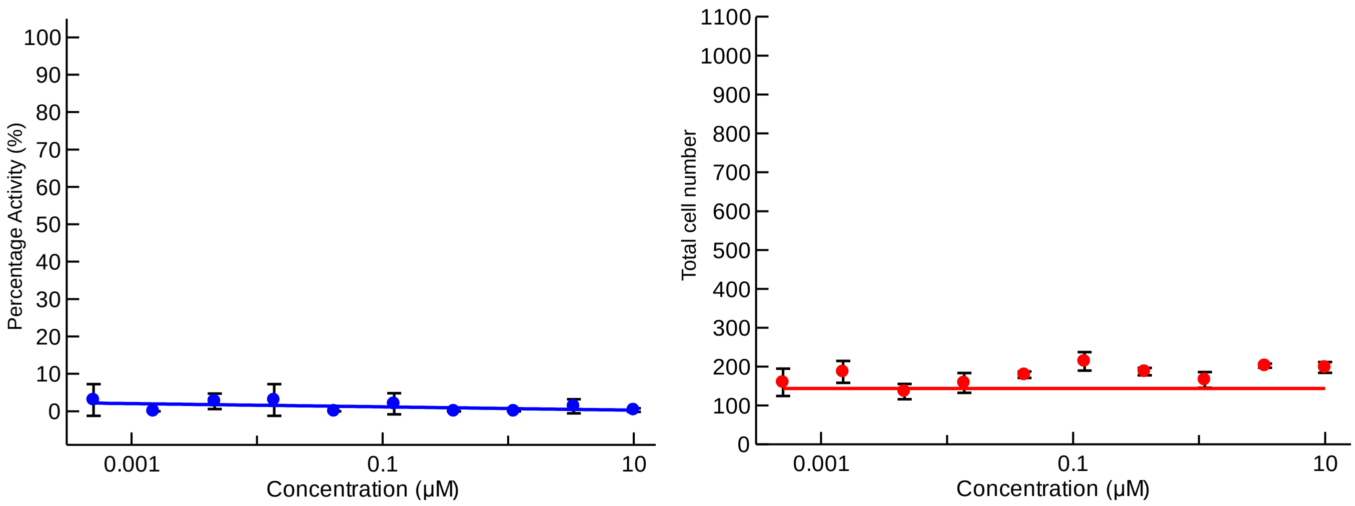

(**a**) (**b**)

© 2014 by the authors; licensee MDPI, Basel, Switzerland. This article is an open access article distributed under the terms and conditions of the Creative Commons Attribution license (http://creativecommons.org/licenses/by/3.0/).
